# Infant responses to direct gaze and associations to autism: A live eye-tracking study

**DOI:** 10.1177/13623613231203037

**Published:** 2023-10-26

**Authors:** Maja Rudling, Pär Nyström, Giorgia Bussu, Sven Bölte, Terje Falck-Ytter

**Affiliations:** 1 Development and Neurodiversity Lab, Department of Psychology, Uppsala University; 2 Uppsala Child and Babylab, Department of Psychology, Uppsala University; 3 Center of Neurodevelopmental Disorders (KIND), Centre for Psychiatry Research; Department of Women’s and Children’s Health & Stockholm Health Care Services, Karolinska Institutet & Region Stockholm; 4 Autism Research Group (CARG), Curtin School of Allied Health, Curtin University; 5 Child and Adolescent Psychiatry, Stockholm Health Care Services, Region Stockholm

**Keywords:** autism spectrum disorder, direct gaze, infant development, live eye tracking

## Abstract

Being looked at is an important communicative signal, and attenuated responses to such direct gaze have been suggested as an early sign of autism. Using live eye tracking, we examined whether direct gaze elicits different gaze responses in infants at ages 10, 14 and 18 months with and without later autism in real-life interaction. The sample consisted of 169 infants: 35 with elevated likelihood of autism and subsequent diagnosis, 94 without subsequent diagnosis and 40 with typical likelihood of autism. Infants in all groups tended to look more towards the adult’s face shortly after the direct gaze occurred. Neither how much nor how quickly the infants responded to the direct gaze differentiated the without elevated likelihood of autism and subsequent diagnosis and with elevated likelihood of autism and subsequent diagnosis groups. Infants in the typical likelihood group looked more at the face after the direct-gaze event than infants in the two elevated likelihood groups, but this result is tentative. In an exploratory analysis, infants in the elevated likelihood of autism and subsequent diagnosis group looked away quicker from faces with direct gaze than infants in the typical likelihood group, but this measure did not correlate with dimensional autism or differentiate between the two elevated likelihood groups. The current results suggest that attenuated behavioural responses to direct gaze in infancy are neither strong nor specific early markers of autism.

**Lay abstract:**

When other people look directly towards us, we often respond by looking back at them, and such direct-gaze responses are important for establishing eye contact. Atypical eye contact is common in autism, but how and when this aspect of autism develops is not well understood. Here, we studied whether how much and how quickly infants respond to others’ direct gaze is associated with autism in toddlerhood. We did this by measuring direct-gaze responses in a playful social interaction using live eye tracking. The study included 169 infants, of whom 129 had an elevated likelihood of developing autism due to having a first-degree family member with the condition, and 40 with typical likelihood of autism. In the elevated likelihood group, 35 were diagnosed with autism spectrum disorder at 3 years of age, and 94 were not. The results showed that infants in all three groups tended to increase their looking towards the adult’s face after the adult looked directly at them. However, neither how much nor how quickly the infants responded to direct gaze by looking back at the adult reliably differentiated the infants with or without subsequent autism. While infants in the elevated likelihood of autism and subsequent diagnosis group tended to look away quicker from faces with direct gaze than infants in the typical likelihood group, this measure did not differentiate between the two elevated likelihood groups. We interpret the results as supporting the view that atypical direct-gaze responses are not early markers of autism.

## Introduction

Eye contact in humans conveys attentional and emotional information, is a signal of communicative intent and constitutes a basis for gaze following and communicative learning (Csibra & Gergely, 2006; Kleinke, 1986; Senju & Csibra, 2008). Eye contact has also been repeatedly shown to affect both cognitive and behavioural processes (known as the eye-contact effect; for a review, see Senju & Johnson, 2009a). As such, it is an important aspect of human interaction. Atypical eye contact is a common and prominent characteristic of autism spectrum disorder (ASD or autism) and has been regarded as a hallmark expression of the condition since the initial observations of Leo Kanner (1943). Common aspects of atypical eye contact in autistic adults are feelings of aversion to eye contact (Trevisan et al., 2017) and reduced eye contact in social interaction (Kliemann et al., 2010). While some studies have found atypical orienting towards other’s eyes and faces in autistic toddlers (e.g. Chawarska et al., 2010; Moriuchi et al., 2017), there has been conflicting evidence for the presence of atypical eye contact already in infancy (Falck-Ytter et al., 2023; Jones et al., 2014; Young et al., 2009).

An important aspect of establishing eye contact is to identify when others look directly towards us and respond by looking back (Csibra & Gergely, 2006). Others’ direct gaze seems to be an especially salient stimulus already in infancy (Farroni et al., 2003), and already at birth, infants are able to identify others’ direct gaze and preferentially orient towards it (Farroni et al., 2002). Perception of others’ direct gaze has been found to enhance face processing already by 4 months of age (Farroni et al., 2002) and facilitate gaze following by 6 months of age (Senju & Csibra, 2008). A such, direct-gaze processing seems to be important already early in life and atypical responsivity to others’ direct gaze in infancy may have pervading and cascading effects on development, especially if accompanied by other types of atypical attention to social stimuli (Corden et al., 2008). Based on that reasoning, direct-gaze responsivity has been suggested as an early developmental basis for atypical eye contact in autism (Klin et al., 2015; Nation & Penny, 2008; Senju et al., 2008; Senju & Johnson, 2009b). There have been studies suggesting atypical processing of direct gaze in autistic children, both in terms of neural processing and behavioural responses (Falck-Ytter et al., 2015; Grice et al., 2005; Nation & Penny, 2008; Senju et al., 2003; Senju & Johnson, 2009b). Language ability and joint attention in childhood have been found to be related to long-term social and adaptive functioning, communication and independence in autistic adults (Gillespie-Lynch et al., 2012). Furthermore, for autistic children, social communication has been linked to self-reported quality of life (De Vries & Geurts, 2015). As eye contact is important for both joint attention and communication in infancy (Csibra & Gergely, 2006; Kleinke, 1986; Senju & Csibra, 2008), infant direct-gaze responsiveness could be an important aspect of early autistic development with long-term consequences for the individual. Yet, very few studies have investigated direct-gaze responses in relation to autism in infancy, in the prodromal stages of the condition when atypical gaze processing has the potential to affect development more generally.

To our To our knowledge, two studies have investigated neural responsivity to averted versus direct gaze in infants, both using event-related potentials (ERPs). Elsabbagh and colleagues found that infants with elevated likelihood (EL) of developing autism differed from their typical-likelihood peers in terms of ERP responses to direct gaze in both computer-generated static (Elsabbagh et al., 2009, 2012) and dynamic (Elsabbagh et al., 2012) stimuli at ages 6 to 10 months. Regarding neural responses to the dynamic stimuli, the small group of infants who was subsequently diagnosed with ASD did not differentiate between direct and averted gaze, which was contrary to the infants who did not later meet the diagnostic criteria (Elsabbagh et al., 2012). However, in a recent follow-up study by Tye et al. (2022), which included a partly overlapping but larger group of 8-month-old infants with subsequent autism, that group difference was not replicated. Instead of being fundamental to autistic development, neural processing of others’ dynamic gaze towards and away from oneself was suggested as a contributing condition that, together with other relevant features, may skew development towards autism (Tye et al., 2022). Focussing instead on real-life behavioural responses to direct gaze, a study by Nyström and colleagues (2017) investigated infants’ responses to an adult’s direct gaze in a playful, real-life interaction. They found that infants who had an EL of developing autism looked less towards the adult’s face within the first second after the adult looked towards the infant. This pattern was not dependent on whether the infant looked towards the adult’s face or away before the direct-gaze event. These results suggest that on short time scales and in real-life interaction, responses to direct gaze could be an early aspect of autism. Importantly though, in the study by Nyström et al. (2017), information on diagnostic or dimensional autism outcomes was not included in the analysis, and it was therefore not possible to assess how specific the results were to autistic development. Taken together, the results of these three studies point towards the possibility of neural direct-gaze responsivity translating into real-life behaviour already in infancy. If so, this could be a clue to understand the development of atypical eye-contact in ASD.

In this study, we followed up the results of Nyström et al. (2017), using the same real-life setting and task, in an extended but partly overlapping sample. As in the analysis by Nyström et al., we measured how much the infants looked towards an adult’s face shortly after the adult looked towards the infant. However, here we included autistic outcome at ~3 years of age, to be able to assess whether direct-gaze responses during interaction with an adult reliably differentiates the infants who go on to meet the criteria of an ASD diagnosis from those who do not, and if direct-gaze responses are associated to autistic characteristics in toddlerhood. In contrast to the Nyström et al. analysis, we also included a longitudinal analysis to investigate if direct-gaze responses develop differently in infants with and without autism between ages 10 and 18 months, and a measure of latency to respond to others’ direct gaze. Based on the hypothesis of direct-gaze responsivity being a basis of atypical eye contact in autism (Klin et al., 2015; Nation & Penny, 2008; Senju et al., 2008; Senju & Johnson, 2009b), we expected the infants with a subsequent autism diagnosis to differ from non-autistic infants in how *much* and how *soon* they looked towards the adult’s face shortly after the adult looked at them. Because autism represents a continuum that extends beyond the diagnostic boundaries (Constantino & Todd, 2003), we also expected these measures to be associated with dimensional autism characteristics in toddlerhood. Furthermore, because many social behaviours in autism have been shown to decrease in frequency during early development (Ozonoff et al., 2010), we anticipated an increased difference in direct-gaze responses over the course of the first 1.5 years of life between the infants with and without subsequent ASD diagnosis.

## Methods

### Participants

Participants were recruited from the greater Stockholm area as part of a longitudinal study following children with EL of developing autism due to having a first-degree family member with the diagnosis (Early Autism Sweden, EASE, http://www.smasyskon.se). Children in the EL group had at least one older sibling and a first-degree family member with an ASD diagnosis, which was verified through medical records. They were recruited via the EASE project’s website, advertisements, and clinical units. Children in the typical likelihood (TL) group had at least one older sibling and no first- or second-degree family members with autism and were excluded if they themselves met the criteria for an ASD diagnosis at 36 months of age. They were recruited via birth records and advertisements. Children were excluded from the study if they had any known, uncorrected hearing or visual impairments, any known genetic or medical conditions, and if they were born before gestational week 36.

Within the EASE study, participating children visited our lab several times from infancy to early childhood, and at ~36 months they were evaluated for neurodevelopmental conditions based on the *Diagnostic and Statistical Manual of Mental Disorders (4th ed.; DSM*-5), using gold standard instruments by experienced clinicians who were blind to the children’s performances on the 10- to 18-month visits. After that evaluation, we retrospectively divided the children into three groups: Infants with elevated likelihood of autism who subsequently received an autism diagnosis (EL-ASD; *n* = 35, 13 girls), infants with elevated likelihood of autism *without* a subsequent autism diagnosis (EL-notASD; *n* = 94, 47 girls), and infants with TL of autism, without subsequent diagnosis (TL; *n* = 40, 21 girls). In the final sample, 27% of the infants with elevated likelihood received an autism diagnosis. The three groups did not differ significantly in terms of socio-economic status (highest educational level on a four-point scale from primary school to tertiary postgraduate, averaged between parents; EL-ASD: *M* = 3.09, *SD* = 0.76; EL-notASD: *M* = 3.19, *SD* = 0.81; TL: *M* = 3.39, *SD* = 0.72; *F*(2, 110) = 0.91, *p* = 0.405) or age (Table 1; Group differences ~10-month visit: *F*(2, 141) = 0.74, *p* = 0.480; ~14-month visit: *F*(2, 147) = 2.68, *p* = 0.072; ~18-month visit: *F*(2, 135) = 0.82, *p* = 0.444). Specific data on ethnicity were not recorded. One infant in the TL group was excluded owing to having received an autism diagnosis after the age of 18 months. The final sample size was dependent on how many infants had completed both the eye-tracking task and the 36-month follow-up at the time of data extraction (February 2022). That extraction date was not decided based on a particular number of participants, as this analysis is part of an ongoing study. The sample included in this study partly overlapped with the study by Nyström et al. (2017; that study included 65 infants in the EL group, and 24 infants in the TL group).

**Table 1.** Descriptive statistics for each group.

Time point	**EL-ASD**			**EL-notASD**			**TL**		
	*N*	Mean	*SD*	*N*	Mean	*SD*	*N*	Mean	*SD*
**Age (days)**									
**10** **m**	29	312.66	18.44	82	311.91	13.82	33	308.52	15.30
**14** **m**	33	437.67	16.68	81	429.30	16.68	36	431.11	20.09
**18** **m**	30	557.43	14.61	75	555.52	16.89	33	560.85	28.87
**Δ Face preference (%)**									
**10** **m**	29	7.42	12.22	82	7.77	10.93	33	13.73	17.64
**14** **m**	33	7.68	10.50	81	8.12	10.07	36	9.18	10.02
**18** **m**	30	9.19	12.32	75	8.29	8.29	33	12.44	14.81
**Face preference periphery early (%)**									
**10** **m**	29	16.14	13.95	82	16.81	12.05	32	19.63	14.57
**14** **m**	33	15.87	10.78	80	17.76	11.74	36	17.81	12.99
**18** **m**	29	12.55	7.49	75	16.60	12.87	33	17.92	16.50
**Face preference periphery late (%)**									
**10** **m**	29	23.48	16.79	82	24.73	12.60	32	25.28	14.63
**14** **m**	33	21.22	10.64	80	24.39	14.78	36	25.42	14.61
**18** **m**	29	18.87	10.15	75	24.41	11.22	33	23.65	14.06
**Face preference central early (%)**									
**10** **m**	20	60.92	29.25	63	68.11	25.41	21	70.61	24.62
**14** **m**	25	62.81	19.69	58	65.50	26.56	29	69.16	23.26
**18** **m**	18	67.69	21.92	59	66.77	22.96	21	71.13	23.56
**Face preference central late (%)**									
**10** **m**	20	37.20	26.18	63	40.55	18.98	21	41.81	16.91
**14** **m**	25	35.98	19.26	58	41.65	21.31	29	44.43	21.95
**18** **m**	18	36.67	16.10	59	41.01	20.00	21	46.47	21.31
**Face looking latency (ms)**									
**10** **m**	25	1025	225	72	1029	281	30	1016	311
**14** **m**	30	971	237	71	947	226	33	962	247
**18** **m**	24	1012	230	70	1036	234	27	989	276
**Baseline face looking (%)**									
**10** **m**	29	18.38	14.57	82	20.65	11.74	33	18.52	11.99
**14** **m**	33	18.48	12.74	81	19.61	14.19	36	21.71	13.37
**18** **m**	30	15.03	12.22	75	20.28	11.85	33	19.43	14.14

### Ethical considerations

The study was approved by the Regional Ethical Board in Stockholm, Sweden, and conducted in accordance with the 1964 Declaration of Helsinki. Written informed consent was provided by all parents. Community members were not involved in developing the research questions or design of this study.

### Procedure and study task

At ages ~10, 14 and 18 months, the infants visited the laboratory for a full day of assessments and experimental tasks. The task analysed in this article was part of an approximately 10-min long live eye-tracking session, and participants were seated in their parent’s lap approximately 200 cm in front of a real-life test leader. Parents were instructed to sit still and to not influence the infant’s looking behaviour during the session. A five-point-calibration was conducted by directing the infants’ attention to a series of positions in front of them using moving stimuli. The calibration procedure was repeated if necessary.

**Figure 1.**
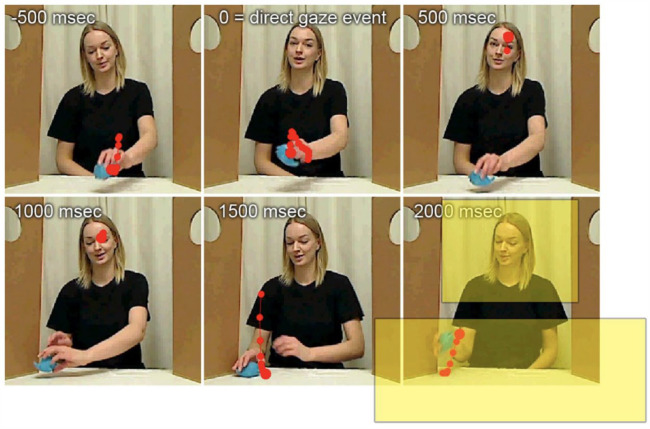
An example of the direct-gaze interactive task. The test leader is playing with a toy. At the direct-gaze event she looks up towards the infant. One participant’s gaze data is superimposed in red dots. Yellow boxes represent the uncropped face and table/toy AOIs, respectively. Reproduced with permission from Nyström et al. (2017).

In the study task, the test leader played with a small toy. They moved the toy across a table in front of them and drew the infant’s attention to the toy by making playful sounds and twice crashing it into a small wall (Figure 1; Full description of procedure in Nyström et al., 2017). During this toy-play, the test leader’s gaze was predominantly directed towards the toy, but intermittently they looked up towards the infant in front of them. The gaze-shifts towards the infant were made to show that the adult was aware of the infant and wanted to share the experience and fun they had with the toy. These gaze shifts, hereafter called direct-gaze events, were also used for marking time zero in each trial. They occurred on average with an interval of 4.35 s, and number of trials per second did not significantly differ between the three groups (Group (EL-ASD, EL-notASD, TL) × Time Point (10, 14, 18 months) ANOVA main effect of group: *F*(2, 423) = 0.36, *p* = 0.839). The direct-gaze interactive task was performed twice during the live eye-tracking session, with two different toys, for approximately 30 seconds each. Because this behaviour was done in a real-life interactive setting each session differed slightly in the number and timing of the trials. The three groups did not differ significantly in terms of number of included trials (Group × Time Point ANOVA main effect of group: *F*(2, 423) = 1.47, *p* = 0.210).

### Data collection, processing and analysis

The infant’s gaze was recorded using a Tobii TX300 eye-tracker, and the test leader’s behaviour using a scene camera. Time-points for when the test leader looked towards the infant was manually identified and integrated with the infant’s gaze data to define the trials. Trials consisted of a baseline period (500 or 100 ms depending on the analysis), and a response period of 2000 ms after the direct-gaze event. The visual scene of the infant was separated into three areas of interest (AOI); one covering the face of the test leader, one covering the toy and table, and one covering the whole scene (Figure 1). Each trial was divided into time-bins of 100 ms each. Trials were excluded if they contained less than 50% gaze data, and inclusion in the analyses required at least 2 valid trials. Missing gaze data in valid trials were linearly interpolated. The average percent data that was interpolated in the included trials was *M* = 19.5, *SD* = 18.7, and this did not differ between the three groups (Group × Time Point ANOVA main effect of group: *F*(2, 423) = 0.45, *p* = 0.638).

The analysis plan was pre-registered in the Open Science Framework (https://doi.org/10.17605/OSF.IO/3EQ5A) after data collection but before any analysis beyond what was included in Nyström et al. (2017). Eye-tracking data were pre-processed in MATLAB (Mathworks Inc., CA, USA) using the Timestudio framework (Nyström et al., 2016) and custom written scripts (which can be found at https://doi.org/10.17605/OSF.IO/3EQ5A). Statistical analyses were performed using SPSS version 27 (IBM Corp., Armonk, NY, USA) and the GAMLj package in Jamovi, version 2.2.5.0 (The Jamovi Project, 2023). Significance tests were two-tailed with an alpha level of *α* *=* 0.05 (if not otherwise stated).

Mixed models were all estimated using restricted maximum likelihood (REML). They included group (EL-ASD, EL-notASD, and TL, with EL-ASD as reference levels) and chronological age at all three time-points (~10, 14 and 18 months) as predictors. This meant that we analysed both the effect of group and the effect of age in the same model. Continuous variables, including age, were centred cluster-wise on participants. Random intercepts were included in all models, and a random slope of age was tested but did not improve the fit of any of the models (Akaike information criterion (AIC) and Bayesian information criterion (BIC)). Model comparisons were performed using AIC and BIC, to find the best-fitting but most parsimonious model in the following order: 1. Main effects of age and group (including random intercept), 2. Pre specified control variables entered individually, 3. Interaction of age by group. Degrees of freedom were estimated using Satterthwaite method. For model comparisons, see Supplemental material Table S1.

To further understand the results of the main analyses, including the non-significant results, we performed equivalent Bayesian analyses of each linear mixed model using Bayesian repeated measures ANOVAs, and Bayesian post hoc group comparisons of the analyses in which we found significant group differences in the main, planned comparisons. The description and results of these exploratory analyses can be found in Supplemental material Appendix S3.

### Measures and analyses

#### Δ face preference

**Figure 2.**
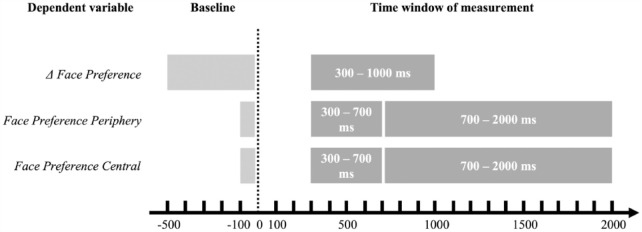
The intervals of the baseline and time windows for measurement of the dependent variables Δ Face Preference, Face Preference Periphery and Central (early and late time windows). See main text for explanations of the intervals. The light grey box shows the respective baseline periods, and the dark grey boxes shows each time window for measurement. Dotted line represents the direct-gaze event, point zero in each trial.

Each trial was divided into a 500-ms baseline before the direct-gaze event (when the test leader looked directly towards the infant), and a 2000-ms measurement window after the direct-gaze event. The baseline value of each trial was then subtracted from each 100-ms time bin of the 2000-ms measurement window. This gave us a timeseries of change in face looking from before to after the direct-gaze event; Δ Face Preference. In the pre-registered analysis plan, we stated that the time windows of interest in which we would aggregate an individual’s data would be based on the time windows used in Nyström et al. (2017; 300–1000 ms after the direct-gaze event). In Nyström et al. (2017), the time windows were defined as the period in which there were significant group differences. After inspecting the plotted timeseries (Figure S1) and confidence intervals we inferred no apparent group differences beyond this pre-specified time window, and Δ Face Preference was therefore calculated by averaging the percent looking towards the face within 300–1000 ms after the direct-gaze event (Figure 2).

#### Face preference periphery and face preference central

**Figure 3.**
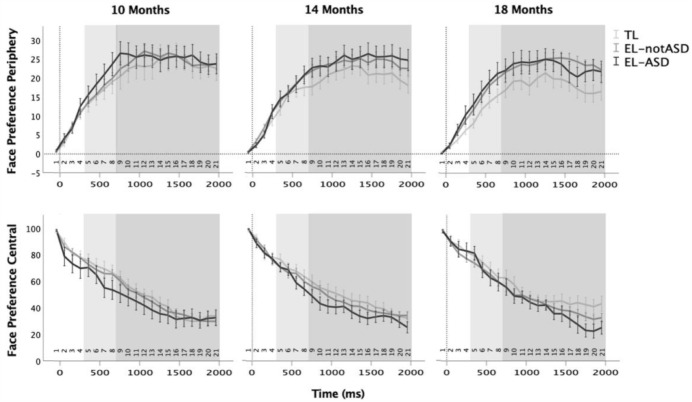
Time series of face preference after the direct-gaze event (0) at each visit (10, 14 and 18 months), for trials in which the test leader’s face was in the infants’ peripheral view (upper row) versus central view (lower row) at the time of the direct-gaze event. Error bars represents standard error of the mean. The light grey box shows the Early Time Window of 300–700 ms after the direct-gaze event (the interval used in Nyström et al., 2017). The dark grey box represents the Late Time Window of 700–2000 ms that was added in the current analysis.

The Δ Face Preference measure included trials independent of where the participant looked before the direct-gaze event. Yet, direct-gaze responses will differ depending on whether the face is in one’s peripheral or central view: If one looks away from the face one can respond to direct gaze by shifting the gaze towards the face, but if one already looks towards the face the only possible change in behaviour is to look away. The Δ Face Preference measure thus captured two qualitatively different behaviours. Therefore, we also separately investigated trials when the infant looked towards the face or towards the table before the direct-gaze onset. In line with the analysis in Nyström et al. (2017), we calculated two measures without baseline correction, instead selected based on behaviour during a shorter baseline period of 100 ms; one using only trials when the infants looked outside of the face AOI before the direct gaze event (< 20% gaze within the face AOI compared to full scene AOI during a baseline period of 100 ms; Face Preference Periphery) and one using trials where the infant looked towards the face AOI at onset of direct gaze (> 80% gaze within the face AOI compared to full scene AOI during 100 ms before; Face Preference Central). The shorter baseline period of 100 ms (versus 500 ms in the Δ Face Preference analysis) was chosen to not lose too many trials due to the stricter inclusion criteria (of percent gaze within the Face AOI; with longer baseline interval more trials will include gaze shifts between AOIs during the baseline interval). Due to the shorter baseline period and different inclusion criteria, slightly different trials were included in these separate analyses than in the combined Δ Face Preference analysis (Face Preference Periphery included 6208 valid trials and Face Preference Central included 1506 trials). See Figure 3 for the time series of each of these measures divided into the three groups and the three time points.

The time-window of interest for calculating average Face Preference Periphery and Face Preference Central was the same as in Nyström et al. (2017) (Early Time Window; 300–700 ms). We also analysed gaze during a Late Time Window of 700–2000 ms (the rest of the trial) to detect potential differences later in the trials (Figure 2). Analyses of this later time window was not pre-registered in the analysis plan.

#### Face looking latency

Latency to look towards the face after the direct-gaze event was defined as the first 100-ms time-bin wherein the infant looked 100% at the face AOI, within a 2000-ms time period after the direct-gaze event (Face Looking Latency). This definition was chosen to keep this measure in line with the measures and processing of data used in Nyström et al. (2017). Face Looking Latency only included trials when the participant did not look at the face before direct-gaze onset, defined as >80% looking at the table AOI for 100 ms before the direct-gaze event (as for the Direct Gaze Periphery measure). For each participant and timepoint (~10, 14 and 18 months) the latency was averaged across trials and multiplied by 100 to convert to milliseconds.

### Associations with dimensional autism characteristics

To test associations with dimensional autism characteristics, we performed bivariate correlations between the direct-gaze response measures at the 10-, 14-, and 18 months visits, and dimensions outcome ratings at the assessment at age 36 months. The outcome measures used were 1) the calibrated severity scores of ADOS-2, ADOS-2 Social Affect, and ADOS-2 Restrictive and Repetitive Behaviour, which is a standardized instrument to measure a child’s social interaction, verbal and nonverbal communication, and restrictive and repetitive behaviours (Lord et al., 2012), and 2) the summed algorithm total scores of ADI-R, which is a semi-structured parental interview aimed to assess a child’s language and communication, reciprocal social interaction, and restrictive, repetitive and stereotypic behaviours and interests (Rutter et al., 2003). To correct for multiple comparisons, we adjusted the alpha level to account for 12 comparisons (Bonferroni, four measures over three time points) to *α* < 0.004. These analyses included only the infants with elevated likelihood of autism, and hence not the TL group.

## Results

Group characteristics and descriptive statistics of each analysis are reported in Table 1.

### Δ Face preference

For all three groups and all three time points, the Δ Face Preference was positive and significantly different from zero, meaning that independent of age, autism likelihood and subsequent ASD diagnosis, all infants were likely to look more towards the adult’s face shortly after the direct-gaze event compared to before (one-sample t-tests, all *p* < 0.003; Bonferroni correction for nine comparisons gives *α* = 0.005).

Accounting for variance explained by age and baseline face looking, the analysis showed that the TL group differed significantly from the EL-ASD group (*t*(157) = 2.01, *p* = 0.046), and the EL-notASD group (*t*(159) = 2.45, *p* = 0.015), but that the two EL groups did not differ significantly from each other (*t*(156) < 0.01, *p* = 0.996). Thus, the infants in the TL group tended to look slightly more towards the adult’s face shortly after the direct gaze event than infants in both the EL groups. Δ Face Preference did not significantly correlate with the dimensional autism outcome measures at any of the three time points.

As noted in the section ‘Methods’, because the Δ Face Preference include both instances when the infants look towards and away from the adult’s face directly before the direct-gaze event, this measure incorporate two contrary types of behaviour. In the previous study by Nyström et al. (2017) the combined Δ Face Preference measure was included due to less statistical power in the divided analysis (below), but in this follow-up analysis with more included participants we focus on the result of the Central/Periphery analysis. The full model specification and result of the Δ Face Preference analysis can therefore be found in Supplemental material (Appendix S1, Figure S1, and Tables S2 and S3).

### Face Preference Periphery and Face Preference Central

For each measure (Face Preference Periphery and Face Preference Central) two linear mixed models (one for each time window) were fitted to the data. The control variable that was tested for inclusion in the model comparisons was test leader identity. For all four analyses, the best-fitting, most parsimonious model was one that included group (EL-ASD, EL-notASD, and TL) and age in days at each time point (10, 14 and 18 months) as fixed effects, but not test leader or the group × age interaction (Face Preference ~ 1 + Age + Group + (1| Participant).

The Face Preference Periphery analysis yielded no significant omnibus or parameter estimates effect of group or age in neither the early or the late time window (omnibus tests: early time window, Group: *F*(2, 162) = 1.42, *p* = 0.245; Age: *F*(1, 267) = 1.16, *p* = 0.283; late time window, Group: *F*(2, 153) = 2.03, *p* = .135; Age: *F*(1, 257) = 1.45, *p* = 0.229, for parameter estimates, see Supplemental material Table S2). For the Face Preference Central analyses, the early time window did not yield a significant effect of group or age (Group: *F*(2, 310) = 1.22, *p* = 0.296; Age: *F*(1, 310) < 0.01, *p* = 0.985) and no significant parameter estimate differences (Supplemental material Table S2). For the late time window, while the omnibus tests were not significant (Group: *F*(2, 132) = 2.31, *p* = 0.103; Age: *F*(1, 161) = 0.32, *p* = 0.573), the parameter estimates of the differences between the TL group and the EL-ASD group were significant (*t*(135) = 2.13, *p* = 0.035). Post hoc tests of EL-notASD–TL (*t*(142) = –0.99, *p* = 0.324) and EL-ASD–EL-notASD (*t*(141) = −1.56, *p* = 0.121) were not significant, indicating that the only significant group difference was between the TL and EL-ASD groups, where the infants with TL of autism looked slightly more at the face specifically within the late time window.

Following the significant group difference between the EL-ASD and TL groups, we investigated the associations between Face Preference Central within the late time window and subsequent dimensional autism outcome in the EL group. Face Preference Central in the late time window was not significantly correlated with any of the ADOS-2 comparison scores or with ADI-*R* (Supplemental material Table S3).

### Face looking latency

For the analysis of Face Latency, the pre-specified control variable that was tested for inclusion in the model comparisons was test leader identity. The model with the lowest AIC and BIC was one that included group (EL-ASD, EL-notASD, and TL) and age in days at each timepoint (10, 14, and 18 months) as dependent variables, but not test leader or the group × age interaction effect (Face Looking Latency ~ 1 + Age + Group + (1| Participant)). In this analysis, neither group nor age was significant in the omnibus tests (Group: *F*(2, 157) = 0.11, *p* = 0.897; Age: *F*(1, 240) < 0.01, *p* = 0.995). Similarly, the fixed-effects parameter estimates showed no significant effects of neither group nor age (Supplemental material Table S2).

The correlation analysis between Face Latency at the three time points and autistic traits at 36 months showed that later gaze shifts towards the adult’s face at the age 18-month time point was associated with higher ADI-R scores at 36 months. However, none of the correlations with the ADOS-2 scores or with ADI-R at the 10- and 14-month visits were approximating significance (Supplemental material Table S3).

### Additional analyses

#### Other neurodevelopmental conditions

The results of the pre-registered analyses indicated that direct-gaze responses are not clearly associated with autism, given the lack of differentiation between the two EL groups. Because there is genetic overlap between several neurodevelopmental conditions (Thapar & Rutter, 2021), we explored if Δ Face Preference was associated with provisional diagnosis of two other neurodevelopmental conditions at 36 months: attention deficit hyperactivity disorder (ADHD) or developmental language disorder. These analyses yielded similar results to the ASD group analysis, with significantly different Δ Face Preference in the TL group compared to the EL groups, but not between the two EL groups, independent of which diagnosis they were partitioned on (Supplemental material Appendix S2).

#### Looking time at the face

As in the Nyström et al. (2017) analysis, we measured how much the infants looked towards the adult’s face throughout the direct-gaze interactive task independent of the looking behaviour of the test leader, to see if the three groups differed in this regard. This was done by the same linear mixed model procedure as in the above analyses, using percent looking towards the face AOI divided by looking time towards the whole scene AOI for the whole direct-gaze interactive task (Percent Face Looking Total ~ 1 + Group + Age + (1| Participant). There was no significant omnibus effect of either group (*F*(2, 162) = 1.35, *p* = 0.261) or age (*F*(1, 262) = 0.78, *p* = 0.378), and no significant group differences for the fixed effects parameter estimates. Similarly, when analysing total looking time towards the whole scene (Percent Scene Looking Total ~ 1 + Group + Age + (1| Participant), there was no significant omnibus effects of either group (*F*(2, 160) = 0.86, *p* = .425) or age (*F*(1, 267) = 0.23, *p* = 0.631) and no significant group differences for the fixed effects parameter estimates. Neither of these analyses were pre-registered in the analysis plan but were performed to control that these factors were unlikely to explain any effects on the outcome variables in the other analyses.

## Discussion

Atypical eye contact is common in autism, and responsivity to others’ direct gaze early in life has been hypothesized as an important driver in the development of this autistic trait and as an early marker of autism (Klin et al., 2015; Nation & Penny, 2008; Senju et al., 2008; Senju & Johnson, 2009b). Yet, no studies have investigated if behavioural responses to direct gaze in infancy is reliably associated with autism. In this study, we used novel live eye-tracking methods to examine whether infants’ looking responses to direct gaze in a real-life interactive situation differentiate those who will subsequently meet the diagnostic criteria of ASD from the infants who will not. Contrary to the above hypothesis, neither how *much* nor how *quickly* the infants looked towards the adult’s face after the direct-gaze event was reliably associated with subsequent ASD diagnosis or dimensional autism outcome in toddlerhood. Instead, infants in all groups tended to look more towards the adult’s face shortly after the adult looked towards them. The three groups also did not differ in how much they looked towards the adult’s face overall, despite the presence of a salient, non-social object in the scene – the colourful toy. Together these findings go against the notion that responsivity to others’ eye contact initiatives during social interaction is markedly different in infants with later autism.

Clear behavioural characteristics of autism are often not apparent until after the first year of life (for a review, see Elsabbagh & Johnson, 2010). We therefore followed the development of direct-gaze responses from the first into the second year of life (at age ~10, 14 and 18 months). We found no effect of age or an interaction between age and group on neither how much nor how quickly the infants responded to others’ gaze directly towards them. This finding was also supported by our follow-up Bayesian analyses (Supplemental material Appendix S3). The lack of a significant effect of age on our measures is contrary to what has been found in the development of social smiles and vocalizations as well as gazes to faces, where the developmental trajectories of infants with subsequent autism diverges more with increasing age from those of non-autistic infants (Ozonoff et al., 2010). Thus, beyond the fact that we found no evidence supporting emergence of behavioural differences in direct-gaze responses in the second year of life, a point when core autistic characteristics typically start to surface, we also did not find that the development of direct-gaze responses follows the same trajectory as other social behaviours observable with the naked eye (social smiles, looks to faces, vocalizations). Instead, our results suggest that behavioural expressions of atypical direct-gaze processing in autism are either not specifically associated with ASD, emerge later in life, or involve other behaviours than those studied here.

The finding that direct-gaze responsivity did not differentiate infants with and without later autism diagnosis is not clearly in line with the previous studies of neural processing of direct gaze, which suggested that processing of gaze shifting towards versus away from the infant might indeed differentiate autistic from non-autistic infants at a group level (Elsabbagh et al., 2012; Tye et al., 2022). Notably, In Tye et al. (2022), neural processing of dynamic direct gaze was not a strong independent predictor of autism on an individual level. It was therefore suggested not as a prerequisite of autism, but rather as a contributing factor to a diffuse pattern of face processing atypicalitites that, taken together, was predictive of autistic development. In both these studies, they measured neural responsivity and not behavioural responses as we did here. It is possible that such neural processing of direct gaze is not translated into differences in behaviour in infancy. Furthermore, whereas those studies of neural processing of direct gaze used static and dynamic computer-generated stimuli, the setting of our behavioural study was real-life interaction. Studies of eye contact and attention to faces in autism have repeatedly found that the type of stimuli and the context of experimental tasks can affect whether autistic participants perform similarly to neurotypical participants (Guillon et al., 2014; Hessels et al., 2018; Speer et al., 2007). To understand real-life effects of autistic development, it is therefore important to increase ecological validity by using naturalistic settings and interactive stimuli as in the current study.

In instances when the infants already looked at the adult’s face before the direct gaze occurred, the infants who later met criteria for an ASD diagnosis tended to look less at the adult’s face during the Late Interval (700–2000 ms after the direct gaze event) compared to the infants with typical likelihood of autism. However, this measure did not correlate with dimensional autism and there were no significant differences between the infants with elevated likelihood of autism who either did or did not later meet criteria for ASD. As such, like with the other results of this study, this finding does not suggest a specific association between direct-gaze responses in infancy and autism. Because the only time window in which we found this significant group difference was not pre-registered, this result is based on an exploratory analysis and should therefore be interpreted with caution. Furthermore, this difference between the TL and EL-ASD groups was not supported by the Bayesian follow-up analyses (Supplemental material Appendix S3). Yet, if replicated, it could suggest that infants who are on the developmental path to autism tend to look away slightly quicker from others’ faces compared to infants without subsequent autism. Potentially, this could mean that others’ faces and direct gaze does not equivalently function as a glue for social looking in autistic and non-autistic development. Autistic children have recently been found to look away quicker from others’ faces compared to non-autistic children (Jónsdóttir et al., 2023), and our finding from infants would be in line with this. If an infant continually looks away from others’ faces too quickly to process the communicative information that often follows direct gaze, and especially if this behaviour continues into childhood, even small effects could have adverse effects on communicative learning, joint attention, and language. These are developmental factors that have been linked to later adaptive functioning, independence, and quality of life in autistic individuals (De Vries & Geurts, 2015; Gillespie-Lynch et al., 2012).

It should be noted that while all but one correlation between direct-gaze responses in infancy and autism characteristics at age 3 were non-significant, we did find a correlation between Face Looking Latency and ADI-R total score at 18 months age. This association remained significant after correcting for multiple correlations (between four autism outcome measures over three time points separately for three direct-gaze response measures). ADI-R is a parental interview measure, capturing aspects of parents’ concerns related to their child’s daily life, which covers partly other aspects than what is captured by clinical observation. Parental concerns have been shown to be informative of later autism outcome in this age group (Sacrey et al., 2015), and it is possible that the correlation we find is indicative of this. Yet, due to the lack of any other significant correlations between direct gaze responses and measures of ADOS and ADI-R at any other time-point, and due to the lack of any significant group differences in how quickly the infants responded to direct gaze, this single significant correlation is difficult to interpret.

We found that infants with an elevated likelihood of developing autism, irrespective of later ASD outcome, looked less towards the adult’s face after the direct-gaze event than their typical-likelihood peers. This finding is in line with what Nyström and colleagues (2017) found, which is unsurprising given the partly overlapping samples. Descriptively, this finding was particularly pronounced at 10 months age, which is also the age previously studied by Nyström et al. (2017). These group differences were further substantiated by the Bayesian follow-up analyses, although only with weak levels of evidence, which suggests that one should be cautious in their interpretation. Based on these findings, however, infant responses to others’ direct gaze may be considered as indicative of genetic predisposition to autism, but not specifically linked to the ASD diagnosis itself or robustly linked to the broader autistic phenotype. A follow-up analysis of associations with provisional ADHD and developmental language disorder diagnoses in toddlerhood (Supplemental material Appendix S2) indicated that direct-gaze responses are likewise not predictive of these diagnoses on a group level. While 3 years is an early age to diagnose both ADHD and developmental language disorder, the results suggest that direct-gaze responses in infancy are also not specifically associated with these common neurodevelopmental conditions, frequently co-occurring with autism. An interpretation of these findings is that responsivity to others’ direct gaze may not be specifically associated with any one, specific neurodevelopmental condition. Rather, it might represent a factor that can skew development further away from the typical path when combined with other more essential, condition-specific developmental factors. For example, in autistic adults, how much one fixates on others’ eyes has been found to be associated with social anxiety, rather than with specifically autistic traits (Corden et al., 2008) or equally associated with both expressions of social anxiety and autism (Hessels et al., 2018).

### Limitations

In any study with potentially important negative results, it is important to consider whether the analyses have enough statistical power. We did find significant effects in our study, with significant group differences between the TL and the EL groups, implying that we had sufficient statistical power to identify effects of a size that are practically relevant. Furthermore, we performed Bayesian follow-up analyses to help interpret the null findings of this study (Supplemental material Appendix S3). The results of these analyses were generally in line with the findings of the main, frequentist analyses except for evidence against an omnibus effect of group on the Δ Face Preference measure in the Bayesian analysis. However, the results of the Bayesian post hoc group difference analysis of this measure were in line with the results of the planned, frequentist group comparisons. Importantly, the frequentist null findings were supported by the Bayesian results, which substantiate their interpretation.

We measured behaviour in a real-life interaction. While this increases the ecological validity of our results, it also means that we sacrificed some control over the stimulus presentation. Reassuringly, neither number of trials per second nor number of valid trials included in the analysis varied significantly between groups. Because our study spanned several years, we had different test leaders during the course of the study (14 in total). To mitigate the effects of individual communicative style on the measures, each test leader was rigorously trained according to clear Standard Operating Procedures and instructional video materials. Test leader identity was also included as potential control variable in all analyses, but this did not improve the fit of any of the models. Neither of these aspects are therefore likely to considerably explain our results.

It is possible that the specific interactive setting used in our study, where the adult sits across from the infant playing alone with a toy, limits the generalizability of our results beyond this specific type of context. For example, Macari and colleagues (2021) did not find differences in attention to adult faces between infants with and without subsequent autism in the context of toy play but did find group differences in the context where touch, speech and gaze were integrated. While that study did not use live eye tracking, the difference in results based on interactive context points towards the importance to follow up our results in other settings. Similarly, other contextual cues such as toy movement or vocalizations could be affecting the gaze behaviour during or study setting, potentially explaining some of the difference in results compared to more controlled experiments (e.g. Elsabbagh et al., 2012; Tye et al., 2022). However, such cues were present both before and after the direct-gaze events, and not specifically temporally linked to them. Also, such contextual cues play an important role in making the interactive setting realistic and engaging for the infant, adding to the ecological validity of our results.

## Conclusion

The results of this live eye tracking study suggest that infants with and without subsequent autism differ very little in their behavioural responses to direct gaze and in how much they look towards faces overall. Instead, we found that irrespective of later autism diagnosis, infants tend to increase their looking towards other’s faces in response to direct gaze. Infant direct-gaze responses were also not robustly associated with the dimensional, broader autistic phenotype. We found this result despite testing several different aspects of direct-gaze responses, following a pre-registered analysis plan. Instead, how much the infants responded to other’s direct gaze seemed linked to having a family history of autism, suggesting a potential role of genes in this behavioural phenotype. Thus, while neural processing of direct gaze in infancy has been found to be linked to the development of autism (Elsabbagh et al., 2012; Tye et al., 2022), these basic differences in social brain functioning do not seem to translate into real-life direct-gaze responses in infancy.

## Supplemental Material

sj-docx-1-aut-10.1177_13623613231203037 – Supplemental material for Infant responses to direct gaze and associations to autism: A live eye-tracking study Supplemental material, sj-docx-1-aut-10.1177_13623613231203037 for Infant responses to direct gaze and associations to autism: A live eye-tracking study by Maja Rudling, Pär Nysträm, Giorgia Bussu, Sven Bälte and Terje Falck-Ytter in Autism
