# Supplementary material for: Infant responses to direct gaze and associations to autism: A live eye-tracking study
Source: Autism. 2023 Oct 26;28(7):1677–89. doi: 10.1177/13623613231203037 (PMC11191372; doi:10.1177/13623613231203037)
Supplement: sj-docx-1-aut-10.1177_13623613231203037 – Supplemental material for Infant responses to direct gaze and associations to autism: A live eye-tracking study [file sj-docx-1-aut-10.1177_13623613231203037.docx]

**Supporting Information**

**Infant responses to direct gaze and associations to autism: A live eye tracking study**

**Appendix S1**

For the Δ Face Preference analysis, the pre-specified control variables to test in the model comparison were test-leader identity and amount of face looking during the baseline period **(**Supporting Information **Table S 1)**. The best-fitting, most parsimonious mixed model was one with Δ Face Preference as dependent variable, and age (in days) at each timepoint (10m, 14m, and 18m), group (EL-ASD, EL-notASD, and TL), and baseline face looking as independent variables (Δ Face Preference ~ 1 + Age + Group + Baseline + (1 | participant)). The omnibus fixed effects tests showed a significant effect of group (*F*(2, 158) = 3.28, *p* = .040), and baseline (*F*(1, 269) = 29.44, *p* < .001), but not of age (*F*(1, 269) = 0.02, *p* = .888). See Supporting Information **Table S 2** for parameter estimates. Planned comparisons of group differences accounting for variance explained by age and baseline face looking showed that the TL group differed significantly from the EL-ASD group (*t*(157) = 2.01, *p* = .046)*,* and the EL-notASD group (*t*(159) = 2.45, *p* = .015), but that the two EL groups did not differ significantly from each other (*t*(156) < 0.01, *p* = .996). The correlation analysis between Δ Face Preference at each time point and autistic traits at ~36 months in the EL group yielded no significant correlations, with an alpha level of *p*=.004 after correcting for 12 comparisons (four measures over three time points; Supporting Information **Table S 3**). The Δ Face Preference analysis included 7967 trials.

**Appendix S2**

We performed a series of exploratory analyses recreating the group variable based on two different diagnostic divisions; whether the child had elevated likelihood of autism and 1) received a provisional ADHD diagnosis at 36 months (ADHD) and 2) received a developmental language disorder diagnosis at 36 months (DLD). The models for these follow-up analyses used the same model specifications as for the Δ Face Preference analysis, with Δ Face Preference as outcome variable (Δ Face Preference ~1 + Age + Group + Baseline + (1 | Participant)).

For the analysis with ADHD division, there was a significant effect of group (*F*(2, 167) = 3.27, *p* = .041) but not of age (*F*(1, 267) = 0.01, *p* = .915), and for the parameter estimates the TL group differed significantly from both the EL-ADHD group (*t*(173) = 2.11, *p* = .036) and the EL-notADHD group (*t*(156) = 2.32, *p* = .022), but the two EL groups did not significantly differ from each other (*t*(177) = 1.83, *p* = .590). Similarly, when dividing the groups on DLD diagnosis there was a significant omnibus effect of group (*F*(2, 165) = 4.15, *p* = .018) but not age (*F*(1, 268) = 0.02, *p* = .896), and the parameter estimates differed significantly between the TL group and the EL-DLD group (*t*(171) = 2.68, *p* = .008), and the EL-notDLD group (*t*(156.63) = 2.22, *p* = .028) respectively, but not between the EL-notDLD and the EL-DLD groups (*t*(174) = 1.37, *p* = .172). These analyses were not pre-registered in the analysis plan.

**Appendix S3**

***Bayesian follow-up analyses***

To help interpret the null findings in the main analysis, we added Bayesian analyses in which the evidence in favour of the null hypotheses could be quantified. Because this was a follow-up test of the hypotheses from the main analyses, we opted to focus on the bayes factor, which quantifies the relative predictive performance of an alternative hypothesis against the null hypothesis (BF_10_; or vice versa: BF_01_). These Bayesian analyses were made using JASP (Version 0.17.1; JASP Team (2023). Each dependent variable (Δ Face Preference, Face Preference Central Early and Late time windows, Face Preference Peripheral Early and Late time windows, and Face Looking Latency) were entered into separate Bayesian repeated measures ANOVAs with timepoint (10, 14, and 18 months) as the repeated factor, and group (EL-ASD, EL-notASD, and TL) as between-subject factor. We used default settings, uniform model priors, and included random slopes of participants for the timepoint (repeated measures) factor. Note that in these analyses we included timepoint instead of exact age.

*Δ Face Preference*

For the Δ Face Preference analysis, the results indicated that there is weak to moderate evidence *against* the hypothesis that there is an effect of group on the Δ Face Preference measure, compared to the null hypothesis (BF_01_ = 2.89, error % = 0.73), strong support for the null hypothesis compared to the model of an effect of timepoint (BF_01_ = 23.47, error % = 1.04), and very strong support for the null hypothesis compared to the model which included both an effect of group and timepoint (BF_01_ = 67.10, error % = 1.24). Further, the (uncorrected) post-hoc comparisons of the three groups showed weak (anecdotal) evidence in support of a difference between the TL and EL-notASD groups (BF_10_ = 1.60, error % = 0.01), and TL and EL-ASD group (BF_01_ = 1.75, error % = 0.02), but moderate support *against* a difference between the EL-notASD and EL-ASD groups, in favour of the null model (BF_01_ = 6.36, error % = 0.07).

*Face Preference Periphery*

The results of the Face Preference Periphery Early analysis yielded strong support for the null model over the model including group (BF_01_ = 5.71, error % = 0.56) and the model including timepoint (BF_01_ = 25.22, error % = 0.81), and extreme evidence for the null model over the model including both group and timepoint (BF_01_ = 139.05, error % = 2.06). The Face Preference Periphery Late analysis likewise resulted in strong support for the null model over the model including group (BF_01_ = 8.98, error % = 0.59), and the model including timepoint (BF_01_ = 27.38, error % = 0.86), as well as extreme evidence in favour of the null hypothesis over the model including both group and timepoint (BF_01_ = 249.74, error % = 1.62).

*The Face Preference Central*

The Face Preference Central Early analysis yielded moderate support for the null hypothesis over the hypotheses that there is an effect of either group (BF_01_ = 5.59, error % = 0.53) or timepoint (BF_01_ = 9.29; error % = 1.07), and very strong evidence against the model including both group and timepoint compared to the null model (BF_01_ = 51.62, error % = 1.19). The Face Preference Central Late analysis yielded moderate support for the null model over the model including group (BF_01_ = 4.88, error % = 0.61), strong evidence against the model including timepoint (BF_01_ = 13.95, error % = 0.74), and very strong evidence against the model including both group and timepoint (BF_01_ = 66.37, error % = 1.46). Because this analysis was the one that indicated a group difference between the TL and EL-ASD groups in the main frequentist analysis, we performed post-hoc comparisons of group differences. These (uncorrected) post-hoc comparisons of the three groups showed moderate support against group differences between the TL group and the two EL groups respectively (the TL and EL-notASD group: BF_01_ = 3.59, error % = 0.02; the TL and EL-ASD group: BF_01_ = 3.31, error % = 0.01) and weak support against a group difference between the EL-notASD and EL-ASD group (BF_01_ = 2.12, error % = 0.02).

*Face Looking Latency*

For the analysis of latency to look towards the adults face after the direct-gaze event the results yielded moderate evidence in favour of the null model compared to the model that included group (BF_01_ = 4.22, error % = 2.72), strong evidence compared to the model that included timepoint (BF_01_ = 11.27, error % = 0.56), and very strong evidence compared to the model that included both group and timepoint (BF_01_ = 47.99, error % = 0.88).

*Conclusions*

Taken together, the results of the Bayesian analyses suggest that there is no support for differences between the groups on any of the direct-gaze response measures, when analysing the three groups together as an omnibus test. This is contrary to the results of the main, frequentist analysis in which we found a significant omnibus effect of group on the Δ Face Preference measure. It is, however, in line with the frequentist null findings of omnibus effects of group for the other measures. We performed post-hoc analyses on the Δ Face Preference and on the Face Preference Central Late measures as we found significant group differences for those measures in the main, frequentist planned comparisons. The Bayesian post-hoc group comparisons for the Δ Face Preference measure supported the finding of the main, frequentist analysis that the two elevated likelihood groups does not differ in terms of the Δ Face Pref. Evidence in favour of group differences between the TL group and the two EL groups (respectively) was only weak, which indicates that there might be reason to be cautious in the interpretation of a difference between the TL and EL groups (which were significant in the main, frequentist analyses). The results of the Bayesian post-hoc comparisons of the Face Preference Central Late Time Window were not in line with the main, frequentist analysis: Whereas the main, frequentist analysis indicated that there is a difference between the TL and EL-ASD groups, the Bayesian analysis yielded moderate evidence against such a difference. Further, and in accordance with the main, frequentist analyses, the Bayesian analyses suggest that there is no difference between the three timepoints in which the infants were tested (­~10-, 14-, and 18 months age).

**Figure S1**

C


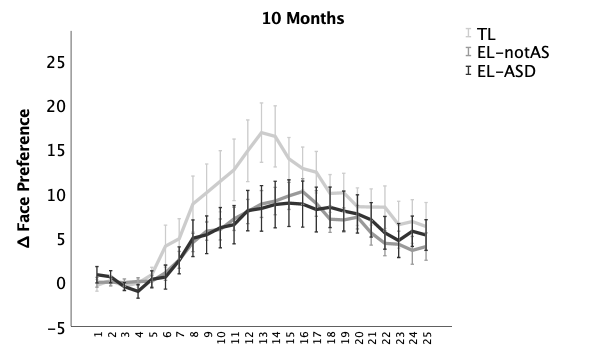

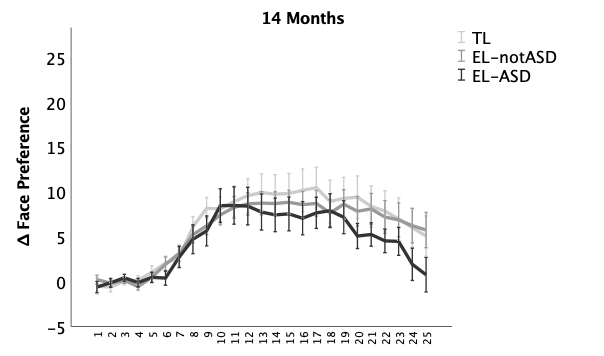

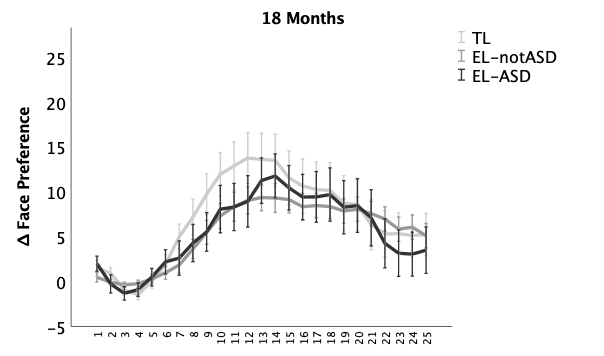

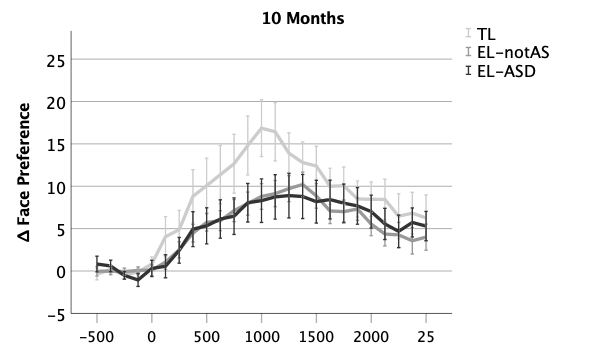

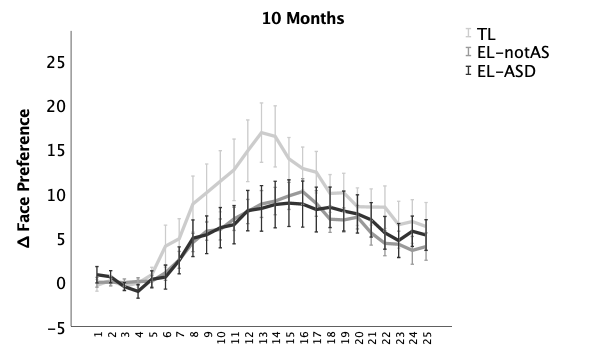

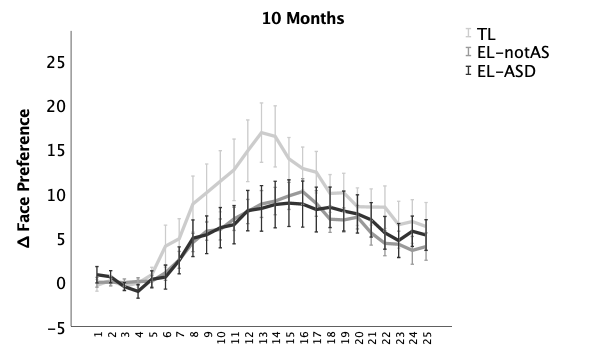

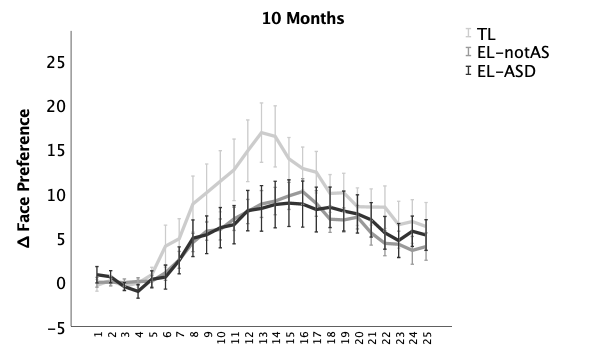

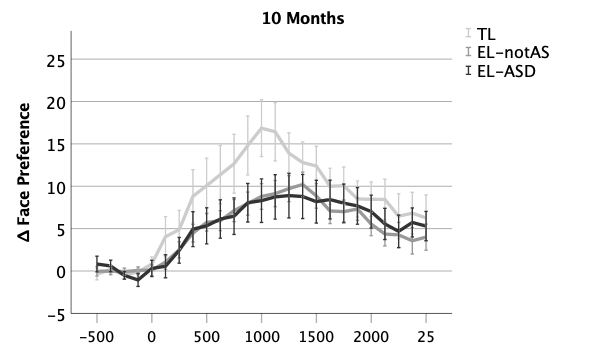

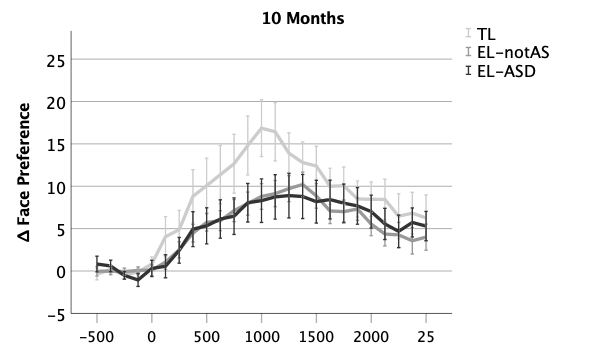

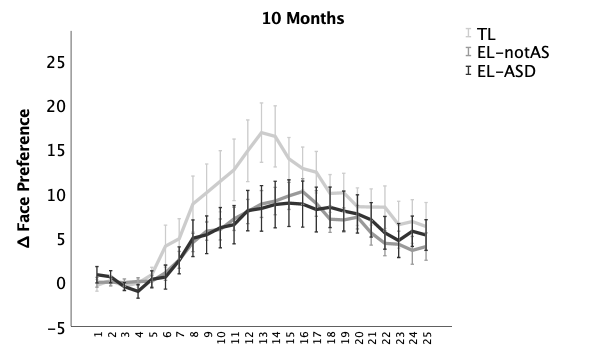

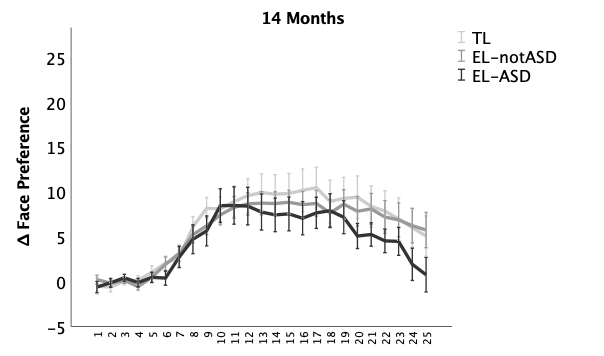

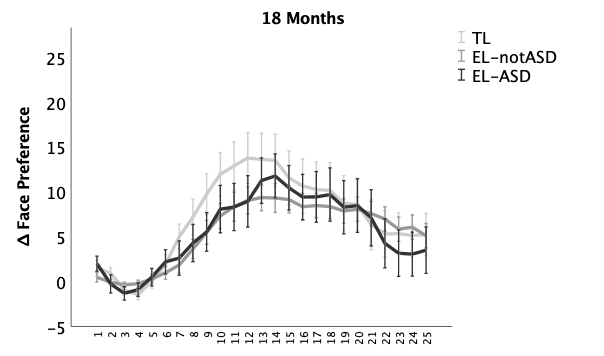

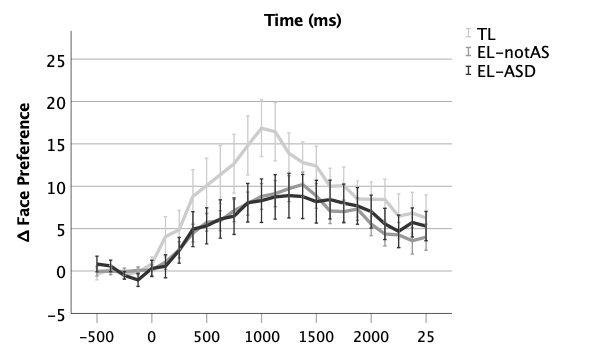

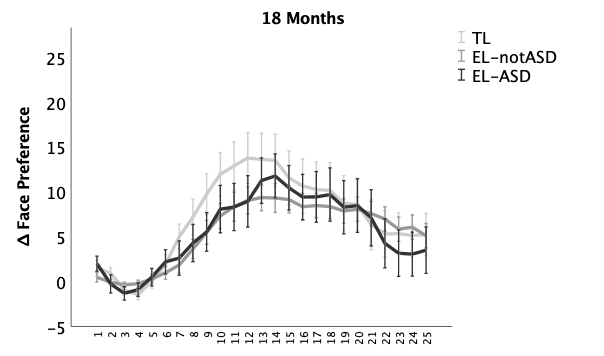

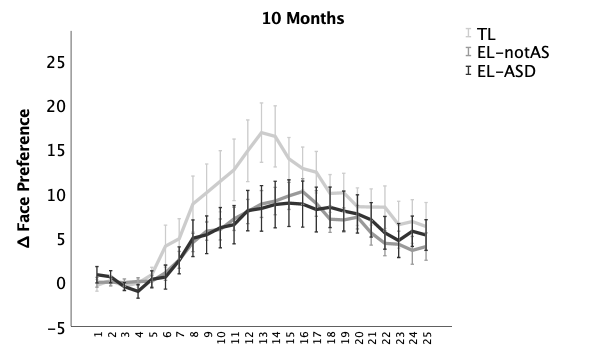


Time series of change in face preference (Δ Face Preference) after the direct-gaze event (0) at each visit (10 months, 14 months, and 18 months). Error bars represents standard error of the mean. The grey box shows the time window used in Nyström et al. (2017) (300-1000 ms after the direct-gaze event), which was also used in the current analysis.

**Table S1. Model comparisons for each linear mixed model analysis**

| **Model Specification** | **AIC** | **BIC** | **LL** | **R^2^ Marginal** | **R^2^ Conditional** |
| --- | --- | --- | --- | --- | --- |
| **Δ Face Preference** |  |  |  |  |  |
| *Base model: Age + Group* | 3376.57 | 3403.39 | -1683.49 | 0.02 | 0.07 |
|  |  |  |  |  |  |
| ***Control Variable: Baseline*** | **3350.42** | **3385.19** | **-1671.36** | **0.07** | **0.16** |
| *Control Variable: Test Leader* | 3379.69 | 3403.30 | -1640.97 | 0.07 | 0.13 |
|  |  |  |  |  |  |
| *Interaction Model: Age * Group* | 3354.09 | 3410.51 | -1677.95 | 0.07 | 0.16 |
| **Face Looking Latency** |  |  |  |  |  |
| ***Base model: Age + Group*** | **1801.50** | **1839.51** | **-901.92** | **< 0.01** | **0.04** |
|  |  |  |  |  |  |
| *Control Variable: Test Leader* | 1809.61 | 1885.98 | -877.59 | 0.06 | 0.09 |
|  |  |  |  |  |  |
| *Interaction Model: Age * Group* | 1805.34 | 1870.26 | -911.35 | < 0.01 | 0.04 |
| **Face Preference Periphery**  **Early Time Window** |  |  |  |  |  |
| ***Base model: Age + Group*** | **3381.39** | **3407.29** | **-1685.46** | **0.01** | **0.22** |
|  |  |  |  |  |  |
| *Control Variable: Test Leader* | 3398.68 | 3412.61 | -1636.60 | 0.05 | 0.25 |
|  |  |  |  |  |  |
| *Interaction Model: Age * Group* | 3384.26 | 3431.75 | -1691.63 | 0.01 | 0.22 |
| **Face Preference Periphery**  **Late Time Window** |  |  |  |  |  |
| ***Base model: Age + Group*** | **3424.98** | **3450.41** | **-1707.02** | **0.02** | **0.25** |
|  |  |  |  |  |  |
| *Control Variable: Test Leader* | 3430.51 | 3442.95 | -1651.77 | 0.07 | 0.25 |
|  |  |  |  |  |  |
| *Interaction Model: Age * Group* | 3426.99 | 3473.85 | -1712.68 | 0.02 | 0.25 |
| **Face Preference Central**  **Early Time Window** |  |  |  |  |  |
| ***Base model: Age + Group*** | **2903.71** | **2921.61** | **-1443.56** | **0.01** | **0.01** |
|  |  |  |  |  |  |
| *Control Variable: Test Leader* | 2922.34 | 2899.40 | -1386.46 | 0.01 | 0.05 |
|  |  |  |  |  |  |
| *Interaction Model: Age * Group* | 2907.60 | 2942.07 | -1448.04 | 0.01 | 0.01 |
| **Face Preference Central**  **Late Time Window** |  |  |  |  |  |
| ***Base model: Age + Group*** | **2784.42** | **2803.56** | **-1384.53** | **0.02** | **0.12** |
|  |  |  |  |  |  |
| *Control Variable: Test Leader* | 2798.38 | 2782.92 | -1328.22 | 0.07 | 0.16 |
|  |  |  |  |  |  |
| *Interaction Model: Age * Group* | 2786.02 | 2822.73 | -1388.37 | 0.02 | 0.13 |

Reporting Akaike Information Criterion (AIC), Bayesian Information Criterion (BIC), Least Likelihood (LL), R^2^ Marginal, and R^2^ Conditional for each model. The best-fitting most parsimonious model was identified comparing AIC and BIC in the order of a three-step hierarchical procedure: 1) Base model wherein the only fixed effects are main effects of Group and Age; 2) Control variables being individually added according to pre-registration; 3) The interaction between Age and Group is added to the best-fitting model from previous steps. The best-fitting, most parsimonious model of each analysis is bolded. R^2^ Marginal, and R^2^ Conditional are informative of the variance explained by the models.

**Table S2. Parameter estimates of the final linear mixed model of each analysis**

|  | **Parameter** | **Estimate** | | | **SE** | | | **95% CI** | | | **df** | | | ***t*** | | ***p*** | | |
| --- | --- | --- | --- | --- | --- | --- | --- | --- | --- | --- | --- | --- | --- | --- | --- | --- | --- | --- |
|  |  |  |  | | | **Lower** | | | **Upper** | | |  | | |  | | |  |
| **Δ Face Preference ~ 1 + Age + Group + Baseline + ( 1 \| Participant )** | | | | | | | | | | | | |  | |  | |  | |
|  | Intercept | 9.22 | 0.66 | | | 7.93 | | | 10.51 | | | 158.03 | | | 13.99 | | | < .001 |
|  | Age | -0.00 | 0.01 | | | -0.01 | | | 0.01 | | | 268.92 | | | -0.14 | | | 0.888 |
|  | EL-notASD - EL-ASD | -0.01 | 1.54 | | | -3.02 | | | 3.01 | | | 156.79 | | | -0.00 | | | 0.996 |
|  | TL - EL-ASD | 3.63 | 1.80 | | | 0.10 | | | 7.16 | | | 157.83 | | | 2.02 | | | 0.046 |
|  | Baseline | -0.33 | 0.06 | | | -0.45 | | | -0.21 | | | 268.92 | | | -5.43 | | | < .001 |
|  |  |  | |  | | |  | | |  | | |  | |  | |  | |
| **Face Looking Latency ~ 1 + Age + Group + ( 1 \| Participant )** | | | | | | | | | |  | | |  | |  | |  | |
|  | Intercept | 9.98 | 0.15 | | | 9.69 | | | 10.27 | | | 157.80 | | | 67.93 | | | < .001 |
|  | Age | -0.00 | 0.00 | | | -0.00 | | | 0.00 | | | 240.45 | | | -0.01 | | | 0.995 |
|  | EL-notASD - EL-ASD | 0.15 | 0.33 | | | -0.49 | | | 0.80 | | | 154.15 | | | 0.46 | | | 0.643 |
|  | TL - EL-ASD | 0.12 | 0.40 | | | -0.67 | | | 0.91 | | | 158.48 | | | 0.29 | | | 0.771 |
|  |  |  | |  | | |  | | |  | | |  | |  | |  | |
| **Face Preference Periphery Early ~ 1 + Group + Age+ ( 1 \| Participant )** | | | | | | | | | | | | |  | |  | |  | |
|  | Intercept | 16.66 | 0.77 | | | 15.15 | | | 18.18 | | | 161.77 | | | 21.54 | | | < .001 |
|  | Age | -0.01 | 0.01 | | | -0.02 | | | 0.00 | | | 266.75 | | | -1.08 | | | 0.283 |
|  | EL-notASD - EL-ASD | 2.08 | 1.81 | | | -1.47 | | | 5.62 | | | 160.26 | | | 1.15 | | | 0.252 |
|  | TL - EL-ASD | 3.55 | 2.12 | | | -0.60 | | | 7.70 | | | 161.57 | | | 1.68 | | | 0.095 |
|  |  |  | |  | | |  | | |  | | |  | |  | |  | |
| **Face Preference Periphery Late ~ 1 + Group + Age+ ( 1 \| Participant )** | | | | | | | | | | | | |  | |  | |  | |
|  | Intercept | 23.44 | 0.83 | | | 21.82 | | | 25.06 | | | 153.02 | | | 28.38 | | | < .001 |
|  | Age | -0.01 | 0.01 | | | -0.02 | | | 0.00 | | | 257.25 | | | -1.21 | | | 0.229 |
|  | EL-notASD - EL-ASD | 3.62 | 1.93 | | | -0.16 | | | 7.41 | | | 151.60 | | | 1.88 | | | 0.063 |
|  | TL - EL-ASD | 3.93 | 2.26 | | | -0.50 | | | 8.36 | | | 152.81 | | | 1.74 | | | 0.084 |
|  |  |  | |  | | |  | | |  | | |  | |  | |  | |
| **Face Preference Central Early ~ 1 + Age + Group + ( 1 \| Participant )** | | | | | | | | | | | | |  | |  | |  | |
|  | Intercept | 66.87 | 1.53 | | | 63.87 | | | 69.87 | | | 310.00 | | | 43.74 | | | < .001 |
|  | Age | 0.00 | 0.02 | | | -0.03 | | | 0.03 | | | 310.00 | | | 0.02 | | | 0.985 |
|  | EL-notASD - EL-ASD | 3.23 | 3.56 | | | -3.76 | | | 10.21 | | | 310.00 | | | 0.91 | | | 0.366 |
|  | TL - EL-ASD | 6.57 | 4.21 | | | -1.69 | | | 14.83 | | | 310.00 | | | 1.56 | | | 0.120 |
|  |  |  | |  | | |  | | |  | | |  | |  | |  | |
| **Face Preference Central Late ~ 1 + Group + Age+ ( 1 \| Participant )** | | | | | | | | | | | | |  | |  | |  | |
|  | Intercept | 40.37 | 1.35 | | | 37.73 | | | 43.01 | | | 132.85 | | | 30.00 | | | < .001 |
|  | Age | 0.01 | 0.01 | | | -0.02 | | | 0.03 | | | 161.39 | | | 0.56 | | | 0.573 |
|  | EL-notASD - EL-ASD | 4.90 | 3.14 | | | -1.25 | | | 11.05 | | | 130.89 | | | 1.56 | | | 0.121 |
|  | TL - EL-ASD | 7.88 | 3.70 | | | 0.62 | | | 15.14 | | | 135.06 | | | 2.13 | | | 0.035 |

**Table S3. Correlations between direct-gaze responses in infancy and autistic traits at 36 months**

|  | **ADOS total** | | |  | **ADOS SA** | | |  | **ADOS RRB** | | |  | **ADI-R** | | |
| --- | --- | --- | --- | --- | --- | --- | --- | --- | --- | --- | --- | --- | --- | --- | --- |
|  | *n* | *r* | *p* |  | *n* | *r* | *p* |  | *n* | *r* | *p* |  | *n* | *r* | *B* |
| ***Δ Face Preference*** | |  |  |  |  |  |  |  |  |  |  |  |  |  |  |
| **10m** | 106 | -0.04 | 0.688 |  | 106 | -0.18 | 0.058 |  | 106 | 0.22 | 0.027 |  | 106 | -0.19 | 0.056 |
| **14m** | 108 | -0.08 | 0.384 |  | 108 | -0.06 | 0.572 |  | 108 | -0.11 | 0.272 |  | 100 | -0.05 | 0.595 |
| **18m** | 102 | -0.04 | 0.664 |  | 102 | 0.04 | 0.706 |  | 102 | -0.20 | 0.046 |  | 92 | -0.04 | 0.715 |
| ***Face Latency*** |  |  |  |  |  |  |  |  |  |  |  |  |  |  |  |
| **10m** | 92 | 0.01 | 0.909 |  | 92 | 0.05 | 0.658 |  | 92 | -0.02 | 0.88 |  | 86 | 0.10 | 0.344 |
| **14m** | 95 | 0.04 | 0.701 |  | 95 | 0.04 | 0.666 |  | 95 | 0.04 | 0.735 |  | 90 | -0.02 | 0.827 |
| **18m** | 92 | 0.06 | 0.573 |  | 92 | 0.03 | 0.764 |  | 92 | 0.06 | 0.546 |  | 83 | 0.31 | 0.004 |
| ***Face Looking Central late time window*** | | | | | |  |  |  |  |  |  |  |  |  |  |
| **10m** | 80 | -0.06 | 0.615 |  | 80 | -0.18 | 0.115 |  | 80 | 0.23 | 0.042 |  | 73 | -0.17 | 0.147 |
| **14m** | 78 | -0.16 | 0.161 |  | 78 | -0.20 | 0.075 |  | 78 | 0.00 | 0.97 |  | 74 | 0.10 | 0.398 |
| **18m** | 75 | 0.01 | 0.955 |  | 75 | 0.07 | 0.542 |  | 75 | -0.23 | 0.043 |  | 69 | -0.08 | 0.533 |

Bonferroni correction for 12 comparisons gives an adjusted alpha level for statistical significance of p < .004
